# Histone Deacetylase Inhibitor SAHA Improves High Salinity Tolerance Associated with Hyperacetylation-Enhancing Expression of Ion Homeostasis-Related Genes in Cotton

**DOI:** 10.3390/ijms21197105

**Published:** 2020-09-26

**Authors:** Shibin He, Yunfei Hao, Qi Zhang, Penghui Zhang, Fengfeng Ji, Hui Cheng, Dong Lv, Yanfeng Sun, Fushun Hao, Chen Miao

**Affiliations:** State Key Laboratory of Cotton Biology, School of Life Sciences, Henan University, Kaifeng 475004, China; sbhe@henu.edu.cn (S.H.); hyf_vin@163.com (Y.H.); 15893785928@163.com (Q.Z.); hui_bio@163.com (P.Z.); 13419987922@163.com (F.J.); ch10475@hotmail.com (H.C.); lvdng@henu.edu.cn (D.L.); yfsun@henu.edu.cn (Y.S.); haofsh@henu.edu.cn (F.H.)

**Keywords:** cotton, high salinity stress, histone deacetylase inhibitor, suberoylanilide hydroxamic acid (SAHA), histone acetylation

## Abstract

Histone acetylation plays an important role in regulation of chromatin structure and gene expression in terms of responding to abiotic stresses. Histone acetylation is modulated by histone deacetylases (HDACs) and histone acetyltransferases. Recently, the effectiveness of HDAC inhibitors (HDACis) for conferring plant salt tolerance has been reported. However, the role of HDACis in cotton has not been elucidated. In the present study, we assessed the effects of the HDACi suberoylanilide hydroxamic acid (SAHA) during high salinity stress in cotton. We demonstrated that 10 μM SAHA pretreatment could rescue of cotton from 250 mM NaCl stress, accompanied with reduced Na^+^ accumulation and a strong expression of the ion homeostasis-related genes. Western blotting and immunostaining results revealed that SAHA pretreatment could induce global hyperacetylation of histone H3 at lysine 9 (H3K9) and histone H4 at lysine 5 (H4K5) under 250 mM NaCl stress, indicating that SAHA could act as the HDACi in cotton. Chromatin immunoprecipitation and chromatin accessibility coupled with real time quantitative PCR analyses showed that the upregulation of the ion homeostasis-related genes was associated with the elevated acetylation levels of H3K9 and H4K5 and increased chromatin accessibility on the promoter regions of these genes. Our results could provide a theoretical basis for analyzing the mechanism of HDACi application on salt tolerance in plants.

## 1. Introduction

Salinity stress is one of the most serious factors limiting plant growth and production. It has been estimated that more than 20% of irrigated land is suffering with high salinity, and the salinized land is becoming more widespread due to climate change and human input [1]. High concentrations of salts in the soil make it difficult for plants to take up water, and excessive salt intake can be toxic to plants, leading to failure in ion homeostasis and growth [2]. As sessile organisms, plants have been forced to evolve the various mechanisms to prevent or alleviate damage caused by high salinity. The key mechanism of salt tolerance is to maintain cellular ion homeostasis by restricting Na^+^ accumulation. Na^+^ exclusion and vacuolar Na^+^ sequestration, mediated by Na^+^/H^+^ antiporters, are two important ways to reduce Na^+^ concentration in the cytoplasm [3]. A well-defined signaling pathway in *Arabidopsis thaliana* for Na^+^ exclusion is the salt overly sensitive (SOS) pathway [3]. The SOS machinery is conserved across different plant species, such as rice, cotton, and tomato [4,5,6]. Salt stress induces a Ca^2+^ signal that triggers SOS3 (a calcium-binding protein) expression, and SOS3 recruits SOS2 (a serine/threonine protein kinase) to the plasma membrane [3]. The SOS3/SOS2 protein kinase complex activates SOS1, a plasma membrane Na^+^/H^+^ antiporter, resulting in Na^+^ export from plant cells [3]. SOS2 also activates vacuolar Na^+^/H^+^ antiporter (NHX1), and the NHX1 is responsible for sequestering Na^+^ into the vacuole. The proton gradient needed for these Na^+^/H^+^ antiporters are driven by the H^+^-ATPase [3]. Overexpression of these ion homeostasis-related genes can enhance the salt stress tolerance in *Arabidopsis* [7], poplar [8], and rice [9].

Recent advances have shown that epigenetic modifications can regulate chromatin structure and affect gene expression, and also play important roles in the plant response to environmental stresses, such as temperature shifts, drought, and flood, as well as high salinity [10]. Histone acetylation is associated with transcriptional activation and is thought to be essential for the response to high salinity stress, such as histone H4 acetylation in tobacco BY2 [11]; acetylation of histone H3 lysine 9 (H3K9ac) and acetylation of histone H4 lysine 5 (H4K5ac) in maize [12]; and H3K9ac, acetylation of histone H3 lysine 14 (H3K14ac), and acetylation of histone H4 lysine 8 (H4K8ac) in *Saccharomyces cerevisiae* [13]. Histone acetylation level is balanced by the antagonistic activities of histone deacetylases (HDACs) and histone acetyltransferases (HATs), which can be used for adjusting plant response to environmental stresses. After NaCl treatment in maize seedlings, the expression of HAT genes *General Control Nondepressible 5* (*GCN5*) and *histone acetyltransferase type B* was elevated, along with the increased global acetylation levels of histone H3 at lysine 9 (H3K9) and histone H4 at lysine 5 (H4K5) [12]. The *gcn5* mutant showed severe growth inhibition under salt stress in *Arabidopsis* [14]. However, some HDACs act as negative regulators in salt stress resistance. The mutants *histone deacetylase 9* (*hda9*), *hda19*, and *hda5/14/15/18/19* exhibited increased tolerance under salt stress in *Arabidopsis* [15,16]. Overexpression of a histone deacetylase 2 gene *PtHDT902* reduced salt stress tolerance in transgenic poplar plants [17]. OsHDA1 was also found to have a negative effect on the transcription activation of *OsSOS1* in rice [18].

The traditional breeding and genetic engineering are widely used to improve salt stress tolerance in plants [19]. Through long time breeding and selection, Sea Rice 86 showed higher tolerance in saline soil [20]. Transgenic plants, such as wheat, rice, and *Arabidopsis*, showed improvements in salt tolerance [21]. However, some plants are difficult to cross or transform. In recent years, exogenous application of small molecules such as abscisic acid, melatonin, and glycinebetaine represent a promising way to increase salt tolerance in plants [22,23,24]. HDAC inhibitors (HDACis), a class of natural and synthetic chemical compounds, can suppress the activity of HDACs and increase histone acetylation level, leading to chromatin decondensation and transcriptional activation [25,26]. HDACis have long been regarded as a class of agents for cancer therapy. Recently, some HDACs, such as cyclo (l-2-amino-8-hydroxamido-suberoyl-aminoisobutylyl-l-phenylalanyl-d-prolyl-), cyclo (l-2-amino-8-oxo-10-oxaundecanoyl-aminoisobutylyl-l-phenylalanyl-d-prolyl-), cylco (l-2-amino-8-thia-10-oxoundecanoyl-aminoisobutylyl-l-phenylalanyl-d-prolyl-), and suberoylanilide hydroxamic acid (SAHA), have also shown the ability to enhance salinity tolerance in *Arabidopsis* and cassava, indicating that there is a potential for improving crop salt tolerance by HDACis [27,28,29].

Cotton (*Gossypium hirsutum* L.) is an important fiber and oil crop in the world [30]. Cotton is classified as a moderately salt-tolerant crop, with a threshold level of 7.7 dS∙m^−1^ and 50% yield decrease at 17 dS∙m^−1^ [31]. There are about 35 million ha of land for cotton fields, and 75% of cotton fields suffer from salt stress [32]. Therefore, high salinity stress is a serious threat to cotton growth and production, especially at germination and seedling stages [1]. Understanding the genetic and epigenetic mechanism of cotton response to salinity may assist in developing strategies for improvements in salt tolerance of cotton. Application of HDACis could help to understand the histone acetylation regulation of the salinity stress in cotton and be a promising option for enhancing salt tolerance in cotton. However, research about histone acetylation involving salt stress is limited in cotton, and whether HDACis could confer salt stress tolerance in cotton is still unclear. In this study, we investigated the function of SAHA, a broad-spectrum of HDACi, during high salinity stress in cotton. We showed that SAHA, with a suitable concentration (10 μM), could relieve high salinity stress in cotton. We confirmed the HDAC inhibitory activity of SAHA in cotton by Western blotting. SAHA treatment strongly elevated global acetylation of H3K9 and H4K5 and upregulated the expression of ion homeostasis-related genes, leading to lower Na^+^ accumulation. Taken together, our results provide some useful information on the salt tolerance in cotton by HDACis application and improve our understanding of histone acetylation regulating abiotic stress in plants.

## 2. Results

### 2.1. SAHA with Appropriate Concentration Improved Tolerance to High Salinity Stress in Cotton

**Figure 1:**
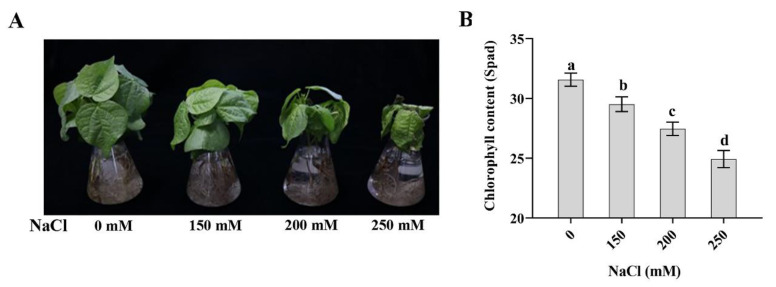
Effects of different NaCl concentrations on growth of cotton seedlings. (**A**,**B**) Phenotype (**A**) and relative chlorophyll content (Spad) (**B**) of three-week-old cotton seedlings treated with different concentrations of NaCl (0/150/200/250 mM) for 5 days. There were three seedlings per bottle. Values are shown as means ± SD of three biological replicates. Different lowercase letters indicate significant difference (*p* < 0.05) between different groups. Tukey’s test after one-way ANOVA was used.

To determine the appropriate NaCl concentration, we transferred three-week-old cotton seedlings to the Hoagland’s solution containing different NaCl concentrations (0/150/200/250 mM), which were further grown for 5 days. As shown in Figure 1A, with the increase of NaCl concentration, the growth inhibition of seedlings became more and more serious. Seedlings growth was most seriously inhibited under 250 mM NaCl treatment for 5 days (Figure 1A). In addition, the chlorophyll content was significantly dropped under 250 mM NaCl treatment (Figure 1B). Therefore, 250 mM NaCl treatment was chosen for further experiments in order to obtain obvious improvement effect of SAHA.

**Figure 2:**
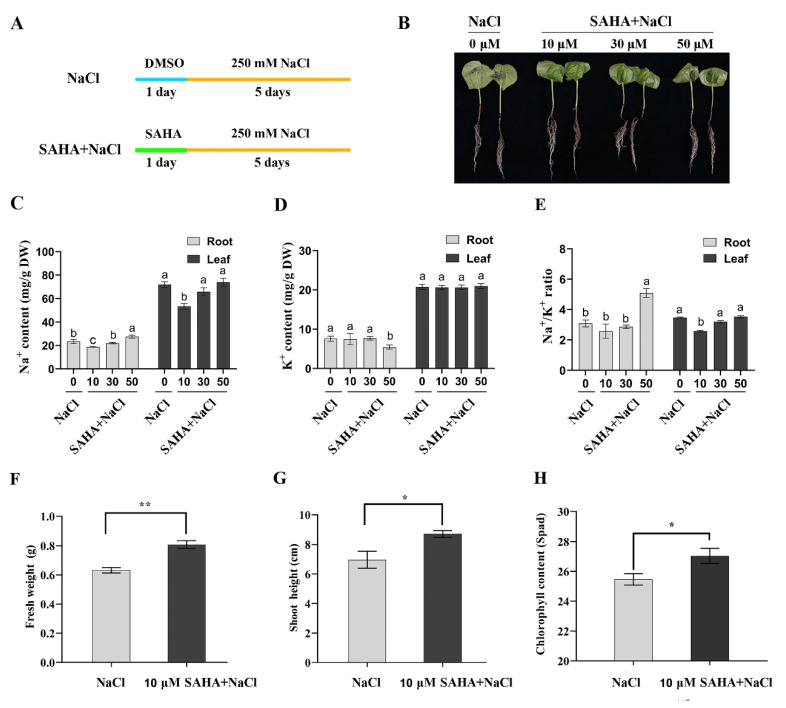
Effects of exogenous application suberoylanilide hydroxamic acid (SAHA) on phenotype and physiology in cotton under 250 mM NaCl stress. (**A**) Experimental scheme of SAHA pretreatment and control groups. The three-week-old cotton seedlings were pretreated with SAHA or DMSO (control) for 24 h, and then subjected to 250 mM NaCl for 5 days. (**B**) Morphological changes of cotton seedlings treated with different concentrations of SAHA (0/10/30/50 μM) and 250 mM NaCl. (**C**–**E**) The effects of different concentrations of SAHA pretreatment on Na^+^ (**C**), K^+^ (**D**) content, and Na^+^/K^+^ ratio (**E**) in roots and leaves after 250 mM NaCl stress for 5 days. Values are shown as means ± SD of three biological replicates. Different lowercase letters indicate significant difference (*p* < 0.05) between different groups. Tukey’s test after one-way ANOVA was used. (**F**–**H**) Fresh weight (**F**), shoot weight (**G**), and relative chlorophyll content (Spad) (**H**) of cotton seedlings pretreated with or without 10 μM SAHA under 250 mM NaCl stress for 5 days. Values are shown as means ± SD of three biological replicates. Student’s *t*-test, * *p* < 0.05, ** *p* < 0.01.

To detect whether SAHA treatment can improve high salinity stress tolerance in cotton and verify the appropriate concentration of SAHA, we pretreated cotton seedlings with different concentrations of SAHA (0/10/30/50 μM) for 24 h and then subjected them to 250 mM NaCl for 5 days (Figure 2A). The 250 mM NaCl treatment without SAHA was used as a control. As shown in Figure 2B, 10 μM SAHA-pretreatment could clearly relieve the symptoms caused by 250 mM NaCl stress, and cotton seedlings grew very well. In addition, only 10 μM SAHA pretreatment for 24 h could slightly improve the growth of cotton seedlings (Figure S1). However, the cotton seedlings pretreated with 30 μM or 50 μM SAHA still exhibited serious symptoms similar to the control group (Figure 2B).

Maintaining Na^+^/K^+^ homeostasis is essential for plant salt tolerance [33,34]. Therefore, we investigated the Na^+^ and K^+^ contents in roots and leaves of cotton under different concentrations of SAHA (0/10/30/50 μM) pretreatment plus 250 mM NaCl stress for 5 days. Cotton roots or leaves under 10 μM SAHA pretreatment accumulated less Na^+^ than control plants (Figure 2C). However, 30 or 50 μM SAHA-pretreated seedlings accumulated a similar amount of Na^+^ with control plants, even with a higher accumulation of Na^+^ in 50 μM SAHA-pretreated cotton roots (Figure 2C). There was a similar K^+^ content in roots and leaves treated with or without SAHA, except for a small decrease of K^+^ content in 50 μM SAHA-pretreated leaves (Figure 2D). Moreover, Na^+^/K^+^ ratio in leaves also decreased under 10 μM SAHA pretreatment (Figure 2E). In addition, the fresh weight, shoot height, and chlorophyll content were also significantly increased under 10 μM SAHA pretreatment (Figure 2F–H). These results indicated that 10 μM SAHA may be a suitable concentration to enhance cotton salt stress tolerance.

### 2.2. SAHA Upregulated the Expression of Ion Homeostasis-Related Genes in Cotton

**Figure 3:**
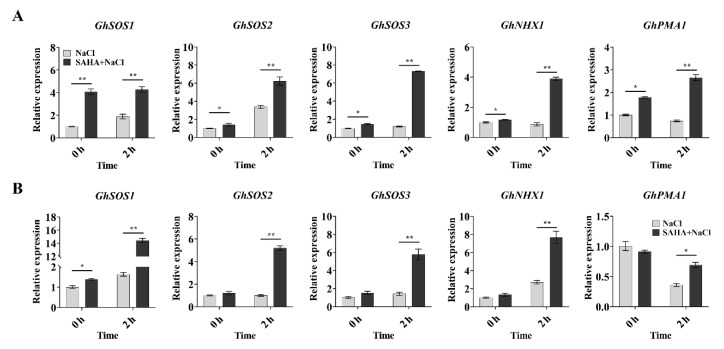
Expression analysis of ion homeostasis-related genes by both 10 μM SAHA pretreatment and 250 mM NaCl stress. (**A**,**B**) Relative expression levels of *GhSOS1*, *GhSOS2*, *GhSOS3*, *GhNHX1*, and *GhPMA1* in roots (**A**) and leaves (**B**) during 250 mM NaCl stress for 0 h and 2 h with/without 10 μM SAHA pretreatment. *GhUBQ7* (*Ubiquitin 7*) was used as the internal control. Values represent the means ± SD of three biological replicates. Student’s *t*-test, * *p* < 0.05, ** *p* < 0.01.

When under salt stress, salt-tolerant plants can usually maintain a low Na^+^/K^+^ ratio in cells by regulating the expression of Na^+^/H^+^ antiporters and of H^+^ pumps [3]. According to the above results, SAHA pretreatment could maintain a low level of Na^+^ under high salinity stress. Moreover, it has been reported that most of the genes were induced in the HDACi-pretreated seedlings after 2 h NaCl stress [27,28]. Thus, we carried out real-time quantitative PCR (qPCR) to detect the expression of some ion homeostasis-related genes, such as *GhSOS1* (CotAD_69068), *GhSOS2* (CotAD_17719), *GhSOS3* (CotAD_19826), *GhNHX1* (CotAD_65716), and *GhPMA1* (encoding plasma membrane proton ATPase, CotAD_43378) for 0 h and 2 h under NaCl treatment. These genes were also highly upregulated in the salt-tolerant genotype of cotton [35]. In our study, SAHA pretreatment could induce the expression of *GhSOS1*, *GhSOS2*, *GhSOS3*, *GhNHX1*, and *GhPMA1* in roots (Figure 3A), and the expression of *GhSOS1* in leaves (Figure 3B) in advance of high salinity stress (0 h). After 2 h NaCl treatment, compared to the control groups, SAHA-pretreated plants also exhibited strongly enhanced expression of these genes in both roots (Figure 3A) and leaves (Figure 3B). These results suggested that the enhanced salt tolerance by SAHA in cotton might have been due to the expression of ion homeostasis-related genes being upregulated, with these genes then being able to perform the function of Na^+^ efflux or Na^+^ sequestration into vacuoles.

### 2.3. SAHA Elevated the Global Histone Acetylation Level in Cotton

**Figure 4:**
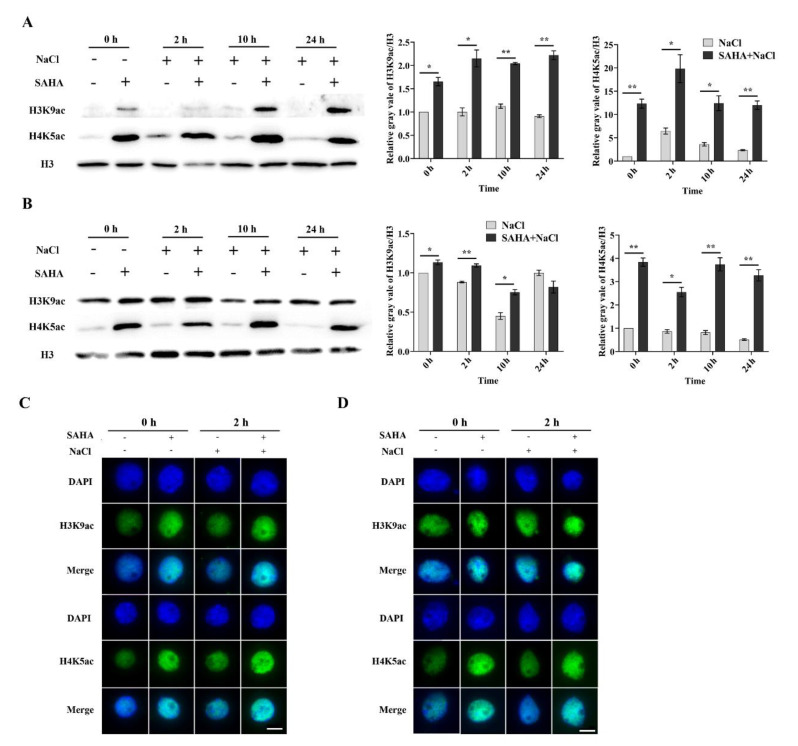
Changes in global histone acetylation level by SAHA pretreatment in roots and leaves under 250 mM NaCl stress. Cotton seedlings were pretreated with/without 10 μM SAHA for 24 h, and then subjected to 250 mM NaCl for 0 h, 2 h, 10 h, and 24 h. (**A**,**B**) Levels of H3K9ac and H4K5ac were detected by Western blotting in roots (**A**) and leaves (**B**). Values represent the means ± SD of three biological replicates. Student’s *t*-test, * *p* < 0.05, ** *p* < 0.01. (**C**,**D**) Levels of H3K9ac and H4K5ac in roots (**C**) and leaves (**D**) were detected by immunostaining. More than 200 nuclei were analyzed. Bar = 5 μm.

Gene activation has a close relationship with histone acetylation. H3K9ac and H4K5ac are usually linked to transcriptional activation [36]. However, it is unknown as to whether SAHA acts as HDACi to change the global acetylation status in cotton. It has been reported that histone acetylation could be significantly increased after NaCl treatment for 24 h [12]. To investigate the HDAC inhibitory activity of SAHA in cotton, we examined the global acetylation levels of H3K9 and H4K5 by Western blotting analysis after NaCl treatment for 0 h, 2 h, 10 h, and 24 h. The acetylation levels of H3K9 and H4K5 were significantly increased in roots (Figure 4A) and leaves (Figure 4B) under SAHA pretreatment. Moreover, the acetylation accumulation was maintained at a high level after additional NaCl treatment (Figure 4A,B). However, both root and leave samples exhibited higher accumulation of H4K5ac when compared to H3K9ac (Figure 4A,B). Furthermore, we performed in situ immunostaining assay to examine the HDAC inhibitory activity of SAHA at the cellular level. Immunostaining analysis showed similar results in roots (Figure 4C) and leaves (Figure 4D), as shown by Western blotting detection. These results suggest that SAHA could act as an HDAC inhibitor and induce genomic histone hyperacetylation in cotton.

### 2.4. SAHA Caused Hyperacetylation of the Promoters of Ion Homeostasis-Related Genes

**Figure 5:**
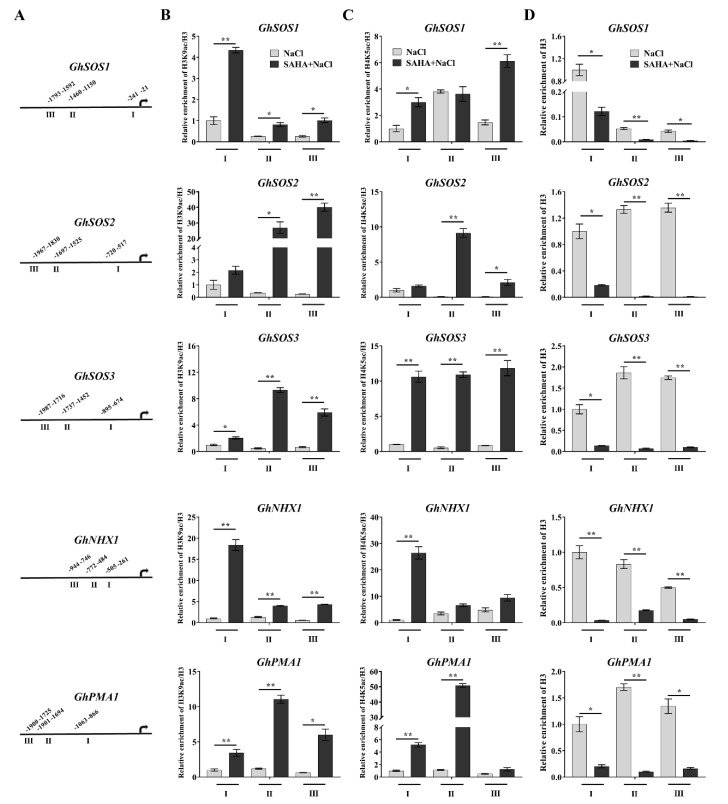
Alterations of H3K9ac, H4K5ac, and H3 on the promoter regions of the ion homeostasis-related genes by SAHA pretreatment in leaves under 250 mM NaCl stress for 0.5 h. (**A**) Detected regions (I, II, and III) of *GhSOS1*, *GhSOS2*, *GhSOS3*, *GhNHX1*, and *GhPMA1* for chromatin immunoprecipitation (ChIP)-qPCR assay. (**B**–**D**) Alterations of the enrichment of H3K9ac (**B**), H4K5ac (**C**), and H3 (**D**) on the promoter regions of *GhSOS1*, *GhSOS2*, *GhSOS3*, *GhNHX1*, and *GhPMA1*. Values represent the means ± SD of three biological replicates. Student’s *t*-test, * *p* < 0.05, ** *p* < 0.01.

Histone acetylation on the promoter regions played an important role in adjustment of gene expression, such as that of H3K9ac and H4K5ac [12]. To examine whether the activated ion homeostasis-related genes were associated with histone hyperacetylation by SAHA-pretreatment, we carried out chromatin immunoprecipitation (ChIP)-qPCR to examine the changes of H3K9ac and H4K5ac on the promoter regions of these genes after NaCl treatment for 0.5 h due to these genes being potentially strongly induced after NaCl treatment for 2 h. The leaves were chosen to perform this assay due to lower Na^+^ accumulation than that of roots (Figure 2E). Three different promoter regions of each gene were selected to perform ChIP-qPCR (Figure 5A). SAHA pretreatment induced a markedly enrichment in the levels of H3K9ac and H4K5ac on almost three promoter regions of the *GhSOS1*, *GhSOS2*, *GhSOS3*, *GhNHX1*, and *GhPMA1* in leaves under 0.5 h salt stress (Figure 5B,C), suggesting that the increased acetylation of H3K9 and H4K5 by SAHA induces upregulation of ion homeostasis-related genes. Remarkably, ChIP with antibody against H3 revealed that there existed deduced H3 level at all promoter regions of these genes (Figure 5D), indicating that SAHA also might induce loss of nucleosomes in these genes, accompanied by the increased levels of H3K9ac and H4K5ac.

### 2.5. SAHA Increased Chromatin Accessibility at Induced Ion Homeostasis-Related Genes

**Figure 6:**
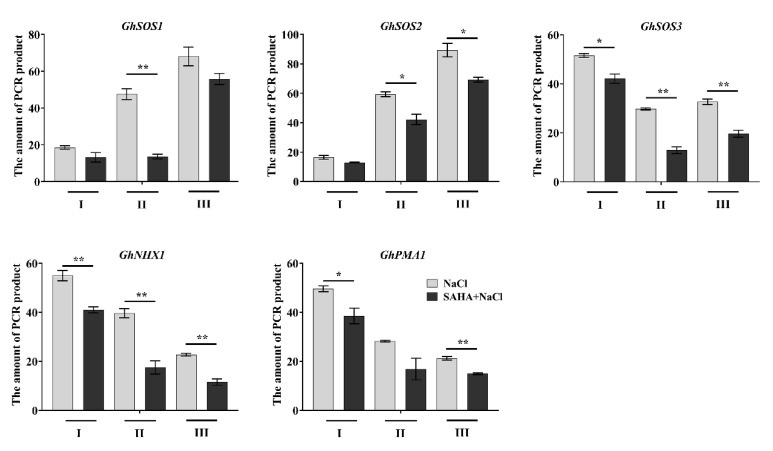
Effects of SAHA pretreatment on the chromatin accessibility of promoter regions of *GhSOS1*, *GhSOS2*, *GhSOS3*, *GhNHX1*, and *GhPMA1* in leaves under 250 mM NaCl stress for 0.5 h. CHART-PCR assay was carried out to measure the sensitivity of the promoter regions of the ion homeostasis-related genes to micrococcal nuclease (MNase). Detected regions (I, II, and III) were same as those used in the ChIP-qPCR assay. The Ct values were converted to the amounts of PCR product according to the standard curve. Values represent the means ± SD of three biological replicates. Student’s *t*-test, * *p* < 0.05, ** *p* < 0.01.

The global change of histone acetylation can also lead to the alternation of chromatin structure [25]. To further confirm the above results (Figure 5D), we used chromatin accessibility real-time PCR (CHART-PCR) to analyze sensitivity of the promoter regions of the ion homeostasis-related genes to micrococcal nuclease (MNase). Compared to the closed chromatin regions, open chromatin regions are more accessible to MNase digestion, and chromatin accessibility is considered to be inversely proportional to the amount of amplified product [25]. As shown in Figure 6, the amount of PCR products in promoters of ion homeostasis-related genes were significantly reduced in SAHA-pretreated plants after 0.5 h salt stress, indicating that these regions were more accessible to MNase and were less compact.

## 3. Discussion

In the present study, we demonstrated that SAHA (a broad-spectrum of HDACi) pretreatment could help to enhance salinity tolerance in cotton. This finding provides useful information for the development of high salinity stress tolerant plants and helps to further advance our understanding of the mechanisms by which histone acetylation regulates plant responses to abiotic stresses.

Maintaining a low concentration of Na^+^ and a high concentration of K^+^ is very important to improve salt tolerance in plants, and this process relies on the expression and activity of ion homeostasis-related genes, including Na^+^/H^+^ antiporter genes and H^+^-ATPase genes [3]. Overexpression of the Na^+^/H^+^ antiporter genes in *Arabidopsis* could maintain a lower Na^+^ concentration to conflict salt stress [7]. Moreover, transgenic sunflower overexpressing PM H^+^-ATPase genes (*HHA4* and *HHA11*) showed higher salinity tolerance by decreasing Na^+^ content [37]. In cotton, overexpression of *GhSOS1* in *Arabidopsis* increased salt stress tolerance through maintaining a low Na^+^/K^+^ ratio [38], and silencing of *GhNHX1* significantly reduced tolerance to salt [39]. Many transcriptomics results have also shown that the expression level of ion homeostasis-related genes could be more rapidly and strongly induced in salt-tolerant varieties than that in salt-sensitive varieties, such as tomato [4], cotton [5], and seashore paspalum [40]. Ion homeostasis-related genes were also found to be more active in the cotton seedlings by SAHA pretreatment in our study (Figure 3), coinciding with relatively less Na^+^ accumulation (Figure 2), which may explain the fact that SAHA-pretreated cotton seedlings have better tolerance to salt stress.

HDACs in plants are separated into three groups: reduced potassium dependency 3/histone deacetylase 1 (RDP3/HDA1), plant-specific histone deacetylase 2 (HD2), and silent information regulator 2 (SIR2) [41]. The RDP3/HDA1 and HD2 groups are Zn^2+^- or Fe^2+^-dependent enzymes, and the RDP3/HDA1 group can be divided into three subgroups (I, II, and IV) [41]. The SIR2 group is a class of NAD^+^-dependent enzymes. In cotton, 29 HDACs were identified, and RDP3/HDA1, HD2, and SIR2 groups contained 18, 7, and 4 members, respectively, indicating that Zn^2+^- or Fe^2+^-dependent HDACs may play a major role in the process of deacetylation [42]. SAHA can competitively bind the catalytic site of HDACs where the Zn^2+^ existed originally and can suppress HDAC enzymatic activity [26]. SAHA was the first HDACi approved by the FDA for clinical use due to its low toxicity [26]. Our results indicated that SAHA is an efficient HDACi that could induce global histone hyperacetylation in cotton (Figure 4), which is consistent with the previous results in cassava [27]. However, in our study, about 10 μM SAHA could improve cotton salt tolerance, while this is 100 μM in cassava [27]. It has been reported that higher concentration of SAHA (5 µM) is toxic to cell culture in *Medicago truncatula* [43]. Maybe SAHA with higher concentration is also toxic to cotton. Moreover, three-week-old cotton seedlings were used in the SAHA pretreatment, while three-month-old cassava were used [27]. It is possible that the optimal concentration is dependent on the plant species and growth stages.

**Figure 7:**
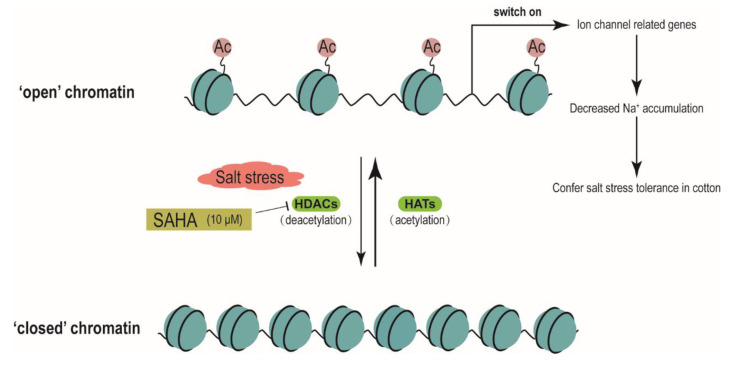
A proposed model for SAHA improvement of salt stress in cotton. Histone acetylation level is balanced by the antagonistic activities of HDACs and HATs. HDACs can remove acetyl groups and lead to a “closed” chromatin structure to repress gene expression. The expression of ion homeostasis-related genes could be turned off due to removal of acetyl groups by recruitment of HDACs to gene promoters. Pretreatment of exogenous SAHA could efficiently inhibit the activity of some HDACs and may lead to global hyperacetylation, which results in an “open” chromatin to activate the expression of ion homeostasis-related genes. Under salt stress, induced expression of ion homeostasis-related genes could enhance the ability to maintain Na^+^/K^+^ homeostasis and alleviate the ion toxicity in cotton.

Reversible histone acetylation and deacetylation play an important role in regulation of gene expression. Histone hyperacetylation, especially at the promoter regions, can relax chromatin structure and lead to transcriptional activation. Under NaCl treatment, upregulation of cell wall expansion-related genes was closely linked to the increased level of H3K9 acetylation on the promoter regions in maize seedlings [12]. It was reported that cyclo(l-2-amino-8-hydroxamido-suberoyl-aminoisobutylyl-l-phenylalanyl-d-prolyl-) activated the expression of *AtSOS1* and *AtSOS3* by increasing the level of histone H4 acetylation in the promoter and coding regions [28]. Trichostatin A, a HDACi, could cause genomic histone hyperacetylation and induce chromatin decondensation on the promoter regions of lateral root development genes to alter the expression of these genes during heat stress in maize seedlings [25]. In our study, SAHA could elevate histone acetylation level, leading to an increased accessibility for transcription factors, which were consistent with the previous results [12,25,28]. ChIP assay with antibodies against H3K9ac and H4K5ac showed that histone acetylation levels on the promoter regions of ion homeostasis-related genes were significantly increased by SAHA pretreatment under salt stress (Figure 5B,C). Moreover, ChIP with anti-H3 and CHART-PCR results suggested that the chromatin accessibility of the promoter regions of ion homeostasis-related genes were increased under SAHA pretreatment (Figure 5D and Figure 6). Our results suggested that SAHA pretreatment could elevate the histone acetylation level and enhance chromatin accessibility to facilitate the expression of these ion homeostasis-related genes, thus leading to lower Na+ accumulation (Figure 7). Moreover, it was reported that the subgroup I HDAC inhibitor is essential for conferring salt tolerance in *Arabidopsis* [15]. SAHA is a broad-spectrum form of HDACi. However, there are many types of HDACi, and different types of HDACi may affect different types of HDAC. It is unclear which type of HDAC is responsible for enhancing salt tolerance in cotton. Therefore, further experiments are needed to using various HDACi to elucidate the relationship between HDACs and salt stress in cotton.

## 4. Materials and Methods

### 4.1. Plant Material and Treatment

Seeds of cotton (*Gossypium hirsutum* L.) inbred line TM-1 were germinated and grown in a greenhouse at normal conditions: 28 °C, 14 h photoperiod, and 70% humidity. Three-week-old cotton seedlings were subjected to suberoylanilide hydroxamic acid (SAHA) for 24 h, and then transferred to the NaCl solution for 2 h, 10 h, or 24 h. SAHA (H1388) was purchased from Tokyo Chemical Industry (Tokyo, Japan) and dissolved in dimethyl sulfoxide (DMSO); DMSO was used as the control for all assays.

### 4.2. Measurement of Growth Parameters, and Na^+^ and K^+^ Contents

The leaf fresh weight and shoot height c were determined after 5 days of NaCl treatment, as described by Tan et al. [44]. The relative chlorophyll contents were made using a SPAD-502 instrument (Konica-Minolta, Tokyo, Japan) between 9:00 a.m. and 10:00 a.m. Moreover, for determination of Na^+^ and K^+^ contents, we dried cotton samples for 72 h in an oven at 70 °C, weighted and ground them into a fine power, and then digested them with 10 mL of a mixture of HNO_3_/HClO_4_ (83:17, *v*/*v*) overnight. Contents of Na^+^ and K^+^ were determined using a Hitachi Z-2000 atomic absorption spectrophotometer (Hitachi, Tokyo, Japan).

### 4.3. RNA Extraction, cDNA Synthesis, and qPCR Analysis

Total RNA was isolated using an RNAprep pure Plant Kit (TIANGEN, Beijing, China), cDNA was synthesized from 1 μg of total RNA with HiScript II 1st Strand cDNA Sythesis Kit (Vazyme, Nanjing, China), and qPCR was performed with ChamQ Universal SYBR qPCR Master Mix (Vazyme, Nanjing, China) with a LightCycler480 instrument (Roche, Basel, Switzerland), using the following profile: 95 °C for 5 min, and then 45 cycles at 95 °C for 10 s, 60 °C for 10 s, and 72 °C for 10 s. *GhUBQ7* (*Ubiquitin 7*) was used as the internal control. The primers used for gene expression in this study are detailed in Table S1. The expression values were calculated using the Schmittgen and Livak method, and the formula is as follows: 2^−ΔΔ*C*^_T_ = 2-[(*C*_T_ of ion homeostasis-related gene—*C*_T_ of *GhUBQ7*) sample A—(*C*_T_ of ion homeostasis-related gene—*C*_T_ of *GhUBQ7*) sample B)] [45]. SampleB indicated the NaCl-treated group at 0 h for gene expression assay, which was used to normalize the data, and sample A indicated other groups.

### 4.4. Antibodies

The following antibodies were applied for Western blotting, immunostaining, and ChIP experiments. Anti-H3K9ac (07–352), anti-H4K5ac (07-327), and FITC-conjugated goat anti-rabbit IgG (12-507) were purchased from Merck Millipore (Darmstadt, Germany). Anti-H3 (ab1791) was obtained from Abcam (Cambridge, MA, USA) and HRP-conjugated goat anti-rabbit IgG (A21020-1) was obtained from Abbkine (Wuhan, China).

### 4.5. Western Blotting and Immunostaining Assay

Extraction of plant proteins was carried out using an Animal-Plant Total Protein Miniprep Kit (TIANDZ, Beijing, China). Western blotting was performed as previously described [46]. The protein samples were separated by sodium dodecylsulphate polyacrylamide gel electrophoresis and transferred to polyvinylidene fluoride membranes. The membranes were blocked with 5% milk in Tris-buffered saline/Tween for 2 h, incubated with the primary antibodies at 4 °C overnight, and then incubated with HRP-conjugated goat anti-rabbit IgG at 37 °C for 2 h. After washing, the membranes were infiltrated with Lumi-Light Western Blotting Substrate (Roche, Basel, Switzerland) and the images were detected by Tanon 4600 instrument (Tanon, Shanghai, China). Histone H3 served as a loading control. Nucleus preparation and immunostaining of nuclei were performed according to the procedure described by He et al. [46]. Nuclei were spread on a slide, blocked with 3% bovine serum albumin in phosphate-buffered saline at 37 °C for 1 h, incubated with primary antibody at 4 °C overnight, and then incubated with FITC-conjugated goat anti-rabbit IgG at 37 °C for 2 h. Immunostained nuclei were counterstained with 4′,6-diamidino-2-phenylindole. Fluorescent signals were captured separately with appropriate filters using Leica GSL-10 Cytogenetic workstation (Solms, Hessen, Germany) equipped with a Cool Snap HQ2 CCD camera (Photometrics, Tucson, AZ, USA). The mean gray values were measured manually in ImageJ software (https://imagej.nih.gov/ij/).

### 4.6. ChIP Assay

ChIP assay was performed according to Zhang et al. [25]. Briefly, 20 g of leaves were ground in liquid nitrogen, suspended in Tris-buffered saline, filtered through miracloth, and resuspended by sucrose solution. The extracted chromatin was digested into 200–500 bp with MNase at 37 °C for 10 min, and immunoprecipitated with antibodies against H3, H3K9ac, and H4K5ac at 4 °C overnight. A negative control was performed using rabbit serum. Immunoprecipitated nucleosomes were eluted by NaCl solutions and an elution buffer (20 mM Tris-HCl, 5 mM EDTA, 50 mM NaCl, and 1% SDS). Then, the eluted DNA was quantified by qPCR. The sequences of all primer sets used for ChIP are listed in Table S2.

### 4.7. CHART-PCR Assay

CHART-PCR assay was carried out to determine the conformational change of chromatin according to Zhang et al. [25]. This process is similar to ChIP. Extracted chromatin was digested with MNase, and then DNA was purified for qPCR with the same primers used in ChIP assay. Chromatin accessibility is considered inversely proportional to the amount of amplified product.

## 5. Conclusions

Overall, our results suggested that exogenous application of SAHA with relative low concentration (10 μM) elevated the acetylation levels of H3K9 and H4K5 and chromatin accessibility on the promoter regions of ion homeostasis-related genes, and then upregulated the expression of these genes, moreover reducing the Na^+^ content, leading to relief of the damage caused by salt stress in cotton seedlings. This study provides the preliminary results for the application HDACi on the development of salt-tolerant varieties of cotton.

## Supplementary Materials

The following are available online at https://www.mdpi.com/1422-0067/21/19/7105/s1.

## Abbreviations

CHART	Chromatin accessibility
ChIP	Chromatin immunoprecipitation
DMSO	Dimethylsulfoxide
H3K9	Histone H3 lysine 9
H4K5	Histone H4 lysine 5
H3K9ac	Acetylation of histone H3 lysine 9
H4K5ac	Acetylation of histone H4 lysine 5
H4K8ac	Acetylation of histone H4 lysine 8
HAT	Histone acetyltransferase
HD2	Plant-specific histone deacetylase 2
HDAC	Histone deacetylase
HDACi	Histone deacetylase inhibitor
NHX	Na^+^/H^+^ exchanger
PMA	Plasma membrane proton ATPase
qPCR	Real-time quantitative PCR
RDP3/HDA1	Potassium dependency 3/histone deacetylase 1
SIR2	Silent information regulator 2
SOS	Salt overly sensitive
Spda	Chlorophyll relative content
SAHA	Suberoylanilide hydroxamic acid
GCN5	General control nondepressible 5
MNase	Micrococcal nuclease
