# Supplementary material for: Histone Deacetylase Inhibitor SAHA Improves High Salinity Tolerance Associated with Hyperacetylation-Enhancing Expression of Ion Homeostasis-Related Genes in Cotton
Source: Int J Mol Sci. 2020 Sep 26;21(19):7105. doi: 10.3390/ijms21197105 (PMC7582796; doi:10.3390/ijms21197105)
Supplement: Supplementary file 1 [file ijms-21-07105-s001.zip › Supplemental figure.docx]

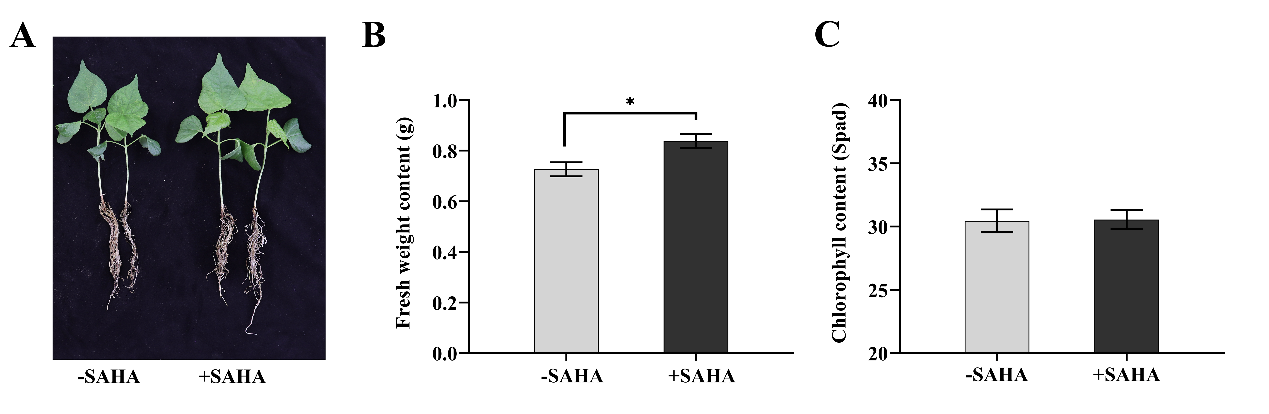


**Figure S1:** Effects of SAHA-pretreatment on growth of cotton seedlings. (A-C) Phenotype (A), fresh weight (B) and relative chlorophyll content (Spad) (C) of cotton seedlings for 5 d with or without 10 μM SAHA-pretreatment for 24 h. Values are shown as means ± SD of three biological replicates. Student’s t-test, *p < 0.05.
