# Supplementary material for: Histone Deacetylase Inhibitor SAHA Improves High Salinity Tolerance Associated with Hyperacetylation-Enhancing Expression of Ion Homeostasis-Related Genes in Cotton
Source: Int J Mol Sci. 2020 Sep 26;21(19):7105. doi: 10.3390/ijms21197105 (PMC7582796; doi:10.3390/ijms21197105)
Supplement: Supplementary file 1 [file ijms-21-07105-s001.zip › Supplemental table.docx]

**Table S1.** Primer sequences used for qPCR.

| Gene name | Forward Primer (5’- 3’) | Reverse Primer (5’- 3’) |
| --- | --- | --- |
| *GhSOS1* | GTGATGGCATTCGACTTTGG | GACCAGCAAGCAGAACCATTT |
| *GhSOS2* | GCATGAGGTTCACTGTGGAAG | AAGTCCGACCCCTTGCTGTAG |
| *GhSOS3* | TTCTTGCTGCTGAAACACCT | AACTCCTCATGCTCGATAAA |
| *GhNHX1* | GCCAGGACTCTTTTGATGAT | AGTGTGTGCTGGAGTTGTAAG |
| *GhPMA1* | TCGTGGTCTGCGGTCATTAG | TGCGAATGGTTTCTGCACTG |
| *GhUBQ7* | GAAGGCATTCCACCTGACCAAC | CTTGACCTTCTTCTTCTTGTGCTTG |

**Table S2.** Primer sequences used for ChIP-qPCR and CHART-PCR.

| Gene name | Region | Forward Primer (5’- 3’) | Reverse Primer (5’- 3’) |
| --- | --- | --- | --- |
| *GhSOS1* | I | ACCTGCTTGCTATCTCCTTCTC | GCATTAGCAGTCATTTTGTCAC |
|  | II | CATCTTACCTAAACCATTCAACAT | CCAAAATAACTGAACGGAATAAG |
|  | III | TATTATCCAAAGTGTCGGTCGG | TTCCTCATCACAACTGTCCAA |
| *GhSOS2* | I | GCAAGAGAGAGAGTTAGAGAAA | GGATTACAAAATCAGTACAAAAGC |
|  | II | TTGCTTTACCTGGTTTGATGAA | AAACCTAGAAGAGAAGTTGCCA |
|  | III | AGCCAAACAGAGATTGCCTAC | AAAGAAACACCTGAACCCAAA |
| *GhSOS3* | I | TTAAGCCACCTTACAAGCCCT | CGTCGGACATCCATACCTCTC |
|  | II | ATGGAACAGTTGGAATGGGACA | GAATCGTTTGATAGTGGTTTGG |
|  | III | CCAAACCACTATCAAACGATTCAC | CTAAACAAAACAAGATTCTCAGGG |
| *GhNHX1* | I | GTCTTGGACAGTTGAAGGTGAA | GCTGAGAAAGCGAAGGAATG |
|  | II | TTCATTCCTTCGCTTTCTCAG | TGCTGCATGATGTTGATTCTC |
|  | III | GTTTTGGAGAATCAACATCAT | CATAAACAGTGAAACATTGGAT |
| *GhPMA1* | I | AACAACAGAGGCGTTCCACAT | GGAAATGTTTTTATGGGAGGG |
|  | II | CTTTGAGAATTGCTGCCCAT | TGGAAGAACATTGATTGGGA |
|  | III | AAAGCCCAGCCTACAATCTCT | GGAAGAACATTGATTGGGATTT |
