# Supplementary figures and images for: Histone Deacetylase Inhibitor SAHA Improves High Salinity Tolerance Associated with Hyperacetylation-Enhancing Expression of Ion Homeostasis-Related Genes in Cotton
Source: Int J Mol Sci. 2020 Sep 26;21(19):7105. doi: 10.3390/ijms21197105 (PMC7582796; doi:10.3390/ijms21197105)

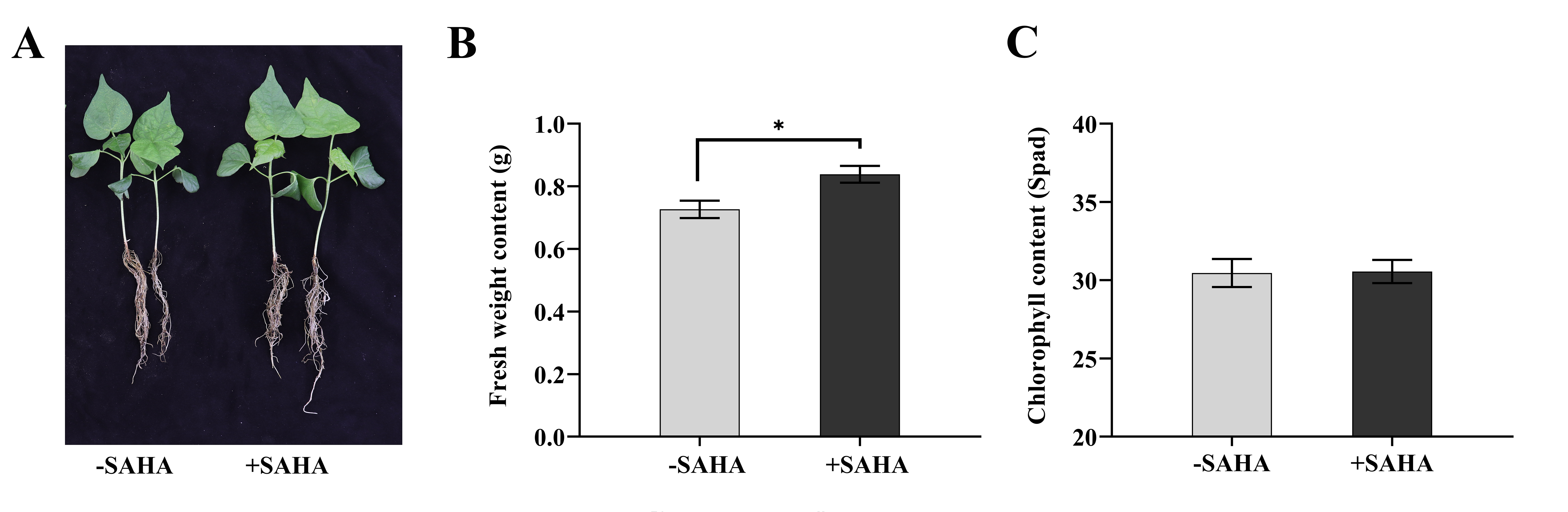

Supplement: Supplementary file 1 [file ijms-21-07105-s001.zip › Supplemental Figure S1.tif]
